# 1351. Use of Mail-Out Sexually Transmitted Infection Test Kits in a Telehealth Pre-exposure Prophylaxis Clinic

**DOI:** 10.1093/ofid/ofab466.1543

**Published:** 2021-12-04

**Authors:** Monica K Sikka, Long Do, Hanifa Ha, Dana Smothers, Christopher Evans, Christopher D Pfeiffer

**Affiliations:** 1 Oregon Health & Science University, Portland, Oregon; 2 Portland VA Medical Center, Portland, Oregon; 3 Portland VA Medical Center/Oregon Health & Science University, Portland, Oregon; 4 VA Portland Health Care System, Portland, Oregon

## Abstract

**Background:**

Standard of care for patients receiving pre-exposure prophylaxis (PrEP) for human immunodeficiency virus (HIV) includes HIV screening and testing for sexually transmitted infections (STIs) at all sites of potential exposure every three months. We implemented a provider and pharmacist telehealth based PrEP program as part of the HIV, Hepatitis Specialty Telehealth Access Resource (H-START) Collaborative. Due to the COVID-19 pandemic and care via telehealth, we had limited ability to collect pharyngeal or rectal swabs in clinic. We created mail-out kits including swabs and instructions for self-collection to test for rectal and pharyngeal *Neisseria gonorrhea* and *Chlamydia trachomatis*.

**Methods:**

Kits were mailed out to patients between June 2020 and May 2021. Providers first confirmed patient comfort with self-swab collection during telehealth appointments. Kits included: an instruction sheet with visual diagrams for collection, swabs with appropriate labels; and a pre-paid envelope for patients to mail swabs back to our facility for laboratory testing. Prospective data collection included the date kits were mailed out to patients, the date of kit receipt at our facility and the test result. Charts were retrospectively reviewed to determine treatment completion.

**Results:**

54 self-swab kits were mailed to patients. 53 of the patients were male and the average age was 41.3 years old. 38 (70.3%) swabs were returned. The median time for return of swabs was 21 days (Range 2-289). Of those returned, 5 (13.1%) were positive and all 5 patients were treated for their infection.

**Conclusion:**

Mail-out STI testing was effective in identifying STIs for a telehealth PrEP program and for maintaining standard of care practice during the COVID-19 pandemic. This model may increase rates of testing compliance for care provided via telehealth and decrease rates of STI transmission and complications. Better communication around returning kits in a timely-manner and understanding reasons for non-return warrant further investigation.

**Disclosures:**

**Monica K. Sikka, MD**, **FG2** (Scientific Research Study Investigator) **Christopher D. Pfeiffer, MD, MHS**, **C. difficile Vaccine Trial** (Scientific Research Study Investigator)

